# Supplementary material for: The relationship between health literacy and the adoption of COVID-19 preventive behaviors: A cross-sectional study in Iran
Source: PLoS One. 2024 May 16;19(5):e0299007. doi: 10.1371/journal.pone.0299007 (PMC11098504; doi:10.1371/journal.pone.0299007)
Supplement: Attachment — Submitted filename: Comment Reviewer The relationship between health literacy and the adoption of COVID-19 preventive.pdf [file pone.0299007.s005.pdf]

The relationship between health literacy and the adoption of COVID-19 preventive behaviors: a cross-sectional study in Iran.

Comments to the Author

Congratulations on the submitted manuscript. The topic is timely and will be of interest to the readers of the journal. However, a few changes are suggested to improve the clarity of this manuscript. I have several recommendations and questions about the manuscript.

No	Page	Descriptions of item	Comment
1.		**Abstract**<br><br>The mean and standard deviation (SD) of the scores of adoption of COVID-19 preventive behaviors and HL among students were 18.18±4.02 out of 25 and 72.14±12.75 out of 100, respectively.<br><br>Adoption of COVID-19 preventive behaviors was less among students with lower levels of HL, male students, students with less physical activity, students with educated mothers, undergraduate students, and smokers.	What are the inclusion and exclusion criteria?<br>Please harmonize the statements. Make confused.<br><br>Please harmonize the statements.
2	11	**Introduction**<br><br>Sajjadi et al.'s<br><br>Azimi et al.'s	Need to explain what is Health literacy.<br><br>Correct this.(Sajjadi et al)
3.	12	**Methodology**<br>among whom 214 were selected by simple random so that the names of 115 all the students were listed and the participants were randomly selected.<br><br>Health literacy for Iranian Adults (HELIA)	Please harmonize this statement.<br><br>This questionnaire was adapted or adopted from where?

4.	15	**Results**<br>**Table 1& 2**<br><br>Findings<br><br>74.8 percent	For the tables, write the symbol<br>% -percent<br>n-frequency<br><br><br>Change to 74.8%
5.	21	**Discussion**	The discussion is too descriptive.<br>How do the findings add to the body of scientific knowledge on the issue?<br>Recommendation:<br>Make the discussion based on subheadings, more clear to the reader.<br>E.g:<br>The relationship between the mother's education level and the adoption of preventive behaviors.
6.	25	**Conclusion**<br><br>Adoption of preventive behaviors against COVID-19 was lower among students with lower levels of health literacy, male students, students with less physical activity, students with educated mothers, undergraduate students, and smokers.	 <br>Please harmonize this statement.
7.		References	Most of the citations are above 5 years.<br>Very good.
