# Supplementary material for: The relationship between health literacy and the adoption of COVID-19 preventive behaviors: A cross-sectional study in Iran
Source: PLoS One. 2024 May 16;19(5):e0299007. doi: 10.1371/journal.pone.0299007 (PMC11098504; doi:10.1371/journal.pone.0299007)
Supplement: Attachment — Submitted filename: Health literacy and the adoption of covid-19 preventive behaviors_review.docx [file pone.0299007.s002.docx]

**The relationship between health literacy and the adoption of covid-19 preventive behaviors: a cross-sectional study in Iran**

Overall comments

Well-written paper. However, some sections need to be revised. The method section should be more concise, and the results should reflect started ways of reporting logistic regressions. The rationale behind health literacy for COVID-19 prevention behaviors should be well outlined in the discussion, especially if medical students have average health literacy. How does this impact health communication with patients or the general population? This manuscript has the potential to impact public health programming. Also, check gramma for errors. See comments below. Good job!

Introduction

1. Line 75-76: there are many types of coronavirus diseases, so it is important to state the specific disease.
2. Line 81: Write COVID-19 in full first
3. Line 99: It may be helpful to indicate the country. (Consider using the third person).
4. Line 112: Was it a “sudden outbreak” worldwide? Consider revising this to more accurate information.

Materials and methods

1. Lines 172- 175: Who were the participants in Mazandaran? Are these different from the 30 university students? Were these two different pilot studies? Were these studies separate from the current study?
2. Lines 183-186: Revise this sentence. Who received the code of ethics?
3. Line 186: What was the nature of informed consent? Written or verbal?
4. Line 190- Enter?
5. While some information in the methods section is helpful, the authors should consider being concise but comprehensive. As it is, it is difficult to follow the methodology logic/flow.

Results

1. Tables 1 and 2: Consider writing the frequency as percentages. E.g. 57.5% rather than 57/5
2. Table 3: It is unclear what chance ratio means.
3. Highlight in Table 3 any significant associations.
4. Use standardized ways of reporting logistic regression results. E.g. Odds ratio, 95%CI,
5. Lines 204-205: The means and standard deviations are not reflected in the tables. Ensure that what is presented in text and table match.
6. n general, the tables are not synchronized. For example, Table 1 has University degree (Bachelor’s / Associate's) _ yet table 3 talks about section as (Associate and master's). Need to be consistent throughout.

Discussion

1. Average literacy levels are based on what measure? What is considered high vs. low?
2. Why is it justifiable that medical students have average health literacy levels, yet they are expected to have higher levels generally?
