# Supplementary material for: The relationship between health literacy and the adoption of COVID-19 preventive behaviors: A cross-sectional study in Iran
Source: PLoS One. 2024 May 16;19(5):e0299007. doi: 10.1371/journal.pone.0299007 (PMC11098504; doi:10.1371/journal.pone.0299007)
Supplement: Attachment — Submitted filename: Response to the comments(Plos one J.).docx [file pone.0299007.s003.docx]

| **Reviewer** | **Response to the comments of the dear Reviewers** |
| --- | --- |
| 1. There are many types of coronavirus diseases, so it is important to state the specific disease. | Done  ( Highlighted in Yellow in the manuscript) |
| 2. Write COVID-19 in full first | Done  ( Highlighted in Yellow in the manuscript) |
| 3. It may be helpful to indicate the country. (Consider using the third person). | Done  ( Highlighted in Yellow in the manuscript) |
| 4. Was it a “sudden outbreak” worldwide? Consider revising this to more accurate information. | Done  ( Highlighted in Yellow in the manuscript) |
| 5. Who were the participants in Mazandaran? Are these different from the 30 university students? Were these two different pilot studies? Were these studies separate from the current study? | The participants in the previous study, conducted in Mazandaran, were people living in Mazandaran- a province in Iran. The designers of this instrument approved its reliability through a pilot study among 30 participants from Mazandaran.  Considering that students were studied instead of ordinary people in the current study, the current research team approved the reliability of this instrument for students by conducting a pilot study among 30 students. Therefore, these two pilot studies are different and separate from each other. |
| 6. Revise this sentence. Who received the code of ethics? | The code of ethics was obtained from the Research and Technology Vice-Chancellor of Esfrain Medical Sciences University by the main researcher ( The Corresponding Author). |
| 7. What was the nature of informed consent? Written or verbal? | Written informed consent was obtained from the students |
| 8. Enter? | Dear Reviewer; As we have mentioned in the manuscript, the variables were entered simultaneously. |
| 9. While some information in the methods section is helpful, the authors should consider being concise but comprehensive. As it is, it is difficult to follow the methodology logic/flow | It was done and some information that seemed to be extra was deleted. |
| 10. Consider writing the frequency as percentages. E.g. 57.5% rather than 57/5 | Done  ( Highlighted in Yellow in the manuscript) |
| 11. It is unclear what chance ratio means. | Done  ( Highlighted in Yellow in the manuscript) |
| 12. Highlight in Table 3 any significant associations. | Done  ( Highlighted in Yellow in the manuscript) |
| 13. Use standardized ways of reporting logistic regression results. E.g. Odds ratio, 95%CI, | Done  ( Highlighted in Yellow in the manuscript) |
| 14. The means and standard deviations are not reflected in the tables. Ensure that what is presented in text and table match. | Qualitative results were reported in the tables, while quantitative results were reported in the text. |
| 15. n general, the tables are not synchronized. For example, Table 1 has University degree (Bachelor’s / Associate's) _ yet table 3 talks about section as (Associate and master's). Need to be consistent throughout. | Done  ( Highlighted in Yellow in the manuscript) |
| 16. Average literacy levels are based on what measure? What is considered high vs. low? | As mentioned in the article, according to the designers of the questionnaire, scores from 0 to 50 are insufficient, 50.1 to 66 not very sufficient, 66.1 to 84 sufficient, and 84.1 to 100 are excellent health literacies.  The average health literacy in the present study was 72, which, according to the research team, was at an average level. In other words, we believed that 72 was at a sufficient level and belonged to the average category. If the average health literacy was between 84.1 and 100, it would be in a good category. Also, if the average health literacy were between 0 and 66, then it would be placed in the weak category. |
| 17. Why is it justifiable that medical students have average health literacy levels, yet they are expected to have higher levels generally? | It was modified in the manuscript:  Considering the effect of the level of education on the level of health literacy of people, and the fact that almost two-thirds of the students in this study were in the first and second years of education, these results could be justified to some extent. |
