# Supplementary material for: The relationship between health literacy and the adoption of COVID-19 preventive behaviors: A cross-sectional study in Iran
Source: PLoS One. 2024 May 16;19(5):e0299007. doi: 10.1371/journal.pone.0299007 (PMC11098504; doi:10.1371/journal.pone.0299007)
Supplement: Attachment — Submitted filename: Responses to Reviewers.docx [file pone.0299007.s004.docx]

| **Additional Editor Comments** | **Point by point Responses to the comments** |
| --- | --- |
| Recommend writing COVID-19 in all caps throughout the text. | OK |
| Be consistent. Will you be using SARS-COV-2 or COVID-19, or both? If using COVID-19, write it in full in the first instance, then put COVID-19 in parenthesis. | OK |
| Move line 87: The definition of Health literacy should be before explaining its effectiveness etc. | Dear Reviewers: In response to your opinion regarding the weakness and inadequacy of the introduction, the research team believes that the introduction should be written according to the keywords in the title of the article. Therefore, in the first paragraphs, we talked about the corona virus and its complications and risk of infection and death. Then it was briefly talked about how to prevent it. Then, we went to health literacy, which is one of the effective factors in preventing Covid-19, and it is related to the adoption of preventive behaviors. Then, the definition of health literacy and the scope of its effects on people's health have been presented. In the following, we have addressed our other key word, students, and we have brought why students were chosen, and also their level of health literacy. Finally, the reason why the present study was done has been summarized. |
| Lines 91-94: Does low health literacy cause diseases? Was there a causal effect? Consider changing the wording to an association or a relationship. | OK |
| Line 90/95: Since you mention studies… provide multiple citations to support the claim. | OK |
| Lines 105-107: need to have more substance. What does average mean? | OK |
| Revise lines 112- 116. This paragraph can be comprehensive by not repeating health literacy in one sentence. | OK |
| Line 116- Provide more information about Esfrain Faculty of Medical Sciences. What City and country? | OK |
| Line 128: Revise this section. Briefly mention the three instruments at the beginning, then proceed to describe the instruments. | OK |
| Line 133-158: The health literacy questionnaire's description is lengthy but can be made clearer. For example, rather than writing out each study that indicates the validity of the scale, simply just state that the questionnaire has been validated by (X, Y, and Z). Also, you could just note that the Likert scale ranges from 5 (completely easy) to 1 (completely difficult). You could also insert Cronbach's alpha coefficient at the beginning next to each item, e.g., Access (α=0.85), instead of repeating the items at the end of the paragraph. | OK |
| 159-175- Condense this information as suggested above. Too long. You don’t have to mention that reliability was measured using a pilot study; simply cite them if describing reliability and validity. | OK |
| Line 188: Suggest revision- Start with a word rather than a numeral. Also, consider stating that the participants completed the survey. “Studied” is a bit vague. What does it mean? | OK |
| Table 2: Bold the “ Behaviors to prevent corona”. Be consistent in the description of COVID-19. Replace “corona” | OK |
| Table 3: What do the figures in bold mean? Include more information as a description at the end of the table. Stick to two or three decimal points throughout. | OK |
| Table 3: Under health literacy is “Enough“ significant? Is there a reason it is bolded? | OK |
| The results section should be the core of the paper. You need to include additional information based on the analysis. What do the odds ratios mean based on the variables? | OK |
| Reviewer #3:   Descriptive title is not suitable for this journal The article is poorly written A proper analysis has not been done | Dear Reviewer; We sought to determine factors influencing the adoption of preventive behaviors against Covid-19. Therefore, to achieve this goal, we used logistic regression, which is a cumulative statistical test that measures the effects of all variables simultaneously. In addition, we believe that if only health literacy was included in the regression model, then our study would be of the correlation type, while it is practically impossible to investigate this relationship without considering other variables. Therefore, in our opinion, a descriptive title is more appropriate. At the same time, we re-examined our analysis. We have strengthened the article as much as possible. |
| Reviewer #4: |  |
| Congratulations on your valuable work. Comments are included to improve your article. Also report health literacy results in the results section. | Dear Reviewer: Health literacy results have already been reported in the results section. |
| In the introduction section, line number 85, first include the definition of health literacy, then include previous studies related to health literacy and healthy behaviors. | Dear Reviewers: In response to your opinion regarding the weakness and inadequacy of the introduction, the research team believes that the introduction should be written according to the keywords in the title of the article. Therefore, in the first paragraphs, we talked about the corona virus and its complications and risk of infection and death. Then it was briefly talked about how to prevent it. Then, we went to health literacy, which is one of the effective factors in preventing Covid-19, and it is related to the adoption of preventive behaviors. Then, the definition of health literacy and the scope of its effects on people's health have been presented. In the following, we have addressed our other key word, students, and we have brought why students were chosen, and also their level of health literacy. Finally, the reason why the present study was done has been summarized. |
| Having or not having the disease of Covid-19 and having or not having the previous experience of suffering from the disease of Covid-19 are not among the entry criteria? | NO |
| In the method section, Cronbach's alpha includes the different dimensions of the health literacy questionnaire. | OK |
| In the method section of line number 180, specify which score is less than 50% and which score is more than 50%. The cut off point is not known. | It was Corrected and strengthened. |
| Information about study 12, 24, and 25 has not been included that these studies were conducted on whom and at what setting and with what tools. Clarify the differences between 2 studies 5 and 28 with the current study. | It was Corrected and strengthened. |
|  |  |
