# Supplementary material for: The relationship between health literacy and the adoption of COVID-19 preventive behaviors: A cross-sectional study in Iran
Source: PLoS One. 2024 May 16;19(5):e0299007. doi: 10.1371/journal.pone.0299007 (PMC11098504; doi:10.1371/journal.pone.0299007)
Supplement: Attachment — Submitted filename: plos one responses to reiewers-third.docx [file pone.0299007.s006.docx]

| **Comments** | **Responses** |
| --- | --- |
| **Reviewer #4:** | The revised parts were highlighted in yellow inside the manuscript. |
| “Health literacy is one of the effective factors in controlling the 45 COVID-19 epidemic and is related to the adoption of preventive behaviors” When you bring this up in the background, it's like the issue is clear and no further research is needed. Revise the sentence. | Tanks so much. It was revised. |
| Line number 86 in the introduction section, define health literacy and describe its dimensions. Of course, it is necessary to mention the dimensions of health literacy and its relationship with behaviors to prevent covid 19.  Include related and similar previous studies in this field in the background of the article and explain the necessity of your study. | Dear Reviewers: the research team believes that the introduction should be written according to the keywords in the title of the article. Therefore, in the first paragraphs, we talked about the coronavirus and its complications and risk of infection and death. Then it was briefly talked about how to prevent it. Then, we went to health literacy, which is one of the effective factors in preventing COVID-19, and it is related to the adoption of preventive behaviors. Then, the definition of health literacy and the scope of its effects on people's health have been presented. In the following, we have addressed our other keyword, students, and we have explained why students were studied, and also their level of health literacy. Finally, the reason why the present study was done has been summarized. |
| Write the time frame of the research. | Ok |
| Explain what tests you used to analyze which variables | Ok |
| Write the discussion more coherently. Write each finding of your study in a paragraph and after you compare it with previous studies, then write your conclusion. | Dear reviewer; First, we started our discussion with the levels of health literacy and the adoption of preventive behavior, and then we analyzed each significant result completely. Please check again. Thanks. |
| **Reviewer #5:** |  |
| The mean and standard deviation (SD) of the scores of adoption of COVID-19 preventive behaviors and HL among students were 18.18±4.02 out of 25 and 72.14±12.75 out of 100, respectively. Please harmonize the statements. Make confused. | Thanks a lot. It was revised. |
| Adoption of COVID-19 preventive behaviors was less among students with lower levels of HL, male students, students with less physical activity, students with educated mothers, undergraduate students, and smokers. Please harmonize the statements. | Adopting preventive behaviors against COVID-19 was lower among students with lower levels of health literacy, male students, students with less physical activity, students with illiterate mothers, undergraduate students, and finally smokers. |
| Introduction Sajjadi et al.'s Azimi et al.'s Need to explain what is Health literacy. Correct this.( Sajjadi et al) | It was revised.  In addition, health literacy was explained in the above lines inside the manuscript. |
| among whom 214 were selected by simple random so that the names of 115 all the students were listed and the participants were randomly selected. Please harmonize this statement. | The population included all male and female students at Esfarayen Medical Sciences University in Iran. The names of all students were listed and 214 participants were randomly selected by simple random sampling. |
| Health literacy for Iranian Adults (HELIA) This questionnaire was adapted or adopted from where? | This questionnaire was designed and psychometrically evaluated by Dr. Montazeri and colleagues in Iran and has the following specifications:  It consists of 33 items in 5 areas including access (6 items), reading skill (4 items), comprehension (7 items), evaluation (4 items), decision making, and use of health information (12 items). Was used. The scoring scale is Likert with 5 choices. In the items related to reading skills; 5 scores were assigned to the completely easy choice, 4 to the easy choice, 3 to neither the easy nor difficult choice, 2 to the difficult choices, and 1 to the completely difficult choice. Also, 5 scores were assigned to always, 4 to most of the time, 3 to sometimes, 2 to rarely, and 1 to never. The scoring method was such that the raw score of each participant in each area was obtained from the algebraic sum of the scores. To convert this score into a range of 0 to 100, the formula of the raw score difference is obtained from the minimum possible raw score divided by the difference between the maximum possible score and the minimum score. Then, to calculate the total score, the scores of all the dimensions (based on the range from zero to 100) are added and divided by the number of dimensions (5 dimensions). Scores from 0 to 50 are considered inadequate health literacy, 50.1 to 66 as insufficient health literacy, 66.1 to 84 as adequate health literacy, and 84.1 to 100 as excellent health literacy (18). This questionnaire has been designed and psychometrically evaluated by Montazeri and colleagues in Iran, and its validity and reliability have been confirmed. The construct validity in the 5 areas was 53.2%, and the reliability of the questionnaire items was determined to be 0.72 to 0.89 by Cronbach's Alpha Coefficient. |
| Table 1& 2(Findings) For the tables, write the symbol % -percent n-frequency 74.8 percent Change to 74.8% | It was revised. |
| The discussion is too descriptive. How do the findings add to the body of scientific knowledge on the issue? | Dear reviewer, we believe that we have done a complete analysis, and all results have been analyzed, compared, and criticized with the existing studies in the discussion part.  To our knowledge, the present study is the first study that evaluated the relationship between different levels of health literacy and the adoption of preventive behaviors against COVID-19 in Iran. Therefore, it can add new findings to the existing scientific knowledge in this field. |
| Recommendation: Make the discussion based on subheadings, more clear to the reader. E.g: The relationship between the mother's education level and the adoption of preventive behaviors. | All these things have been done and each significant relationship has been analyzed and compared. |
| Adoption of preventive behaviors against COVID-19 was lower among students with lower levels of health literacy, male students, students with less physical activity, students with educated mothers, undergraduate students, and smokers. Please harmonize this statement. | Adopting preventive behaviors against COVID-19 was lower among students with lower levels of health literacy, male students, students with less physical activity, students with illiterate mothers, undergraduate students, and finally smokers. |
| References Most of the citations are above 5 years. Very good. | Thanks so much. |
| **Reviewer #6:** |  |
| Line 55: you do not need to mention standard deviation. Just insert the mean score and insert SD in parentheses: 18.18 (4.02) | Thanks a lot, it was revised. |
| Line 95: This seems more correct: University student population in Iran | Thanks a lot, it was revised. |
| Line 96: "Due to the important role, they play as managers and future planners in the country" omit the comma | Thanks a lot, it was revised. |
| Line 100: "Sajjadi et al.'s study showed that one-third of the students had insufficient or not very please for this and other studies mention the sample groups more specific. | Thanks a lot, it was revised. |
| Line 102: " The critical situation of the prevalence of COVID-19 disease can cause negative and positive psycho-social effects in society including the university students [14]" this sentence seems unnecessary. what is the relevance of this to your study? Did you consider these effects in your work? | Yes, we considered it.  We wanted to say that COVID-19 can even affect students. |
| Line 107-110: Was your sample group medical students or from other majors in health sciences? You may mention it here and it seems more reasonable when the participants are from health sciences. | Esfrain Medical Sciences University has only 7 different fields of study including nursing, public health, intelligence, operating room technician, etc. |
| The method section was described thoroughly and in details. Some comments: How did you chose the background variables? | We chose the background variables by studying various studies in the field of health literacy and COVID-19. |
| Line 113-116: "The statistical population was all the male and female students of Esfarayen Faculty of Medical Sciences, North Khorasan, Iran, among whom 214 were selected by simple random so that the names of all the students were listed and the participants were randomly selected." Please re-write this art in proper English writing. | The population included all male and female students at Esfarayen Medical Sciences University in Iran. The names of all students were listed and 214 participants were randomly selected by simple random sampling. |
| Line 131: parents' | Thanks, it was revised. |
| Line 133: what do you mean by "type of COVID-19" | We meant the type of infection, such as O’micron, Gamma, Beta, etc. |
| LINE 179: please insert numbers alphabetically in the beginning of a sentence. | Ok |
| Table 1: what do you mean by "How to get content"- what content? | It was revised. |
| Table 1: it would be much better if you asked participants the weekly amount of their exercise and in some ranges (e.g more than 2 hours, less than 2 hours …) this way it would be more objective. | Thanks a lot, your suggestion is very good, but the study has been completed and it can no longer be done. |
| Table 1: what is the importance of their Covid-19 type? What difference does it make? | We wanted to see whether COVID-19 infection can be effective in preventing re-infection or initial infection. |
| Results: would you please a chart or graph (as supplementary items) that shows the questionnaires items and the frequency of each answers in specific? | Thanks so much, but the results should be mentioned based on the most important goals of the study. |
| Discussion: " These results were consistent with the results of Panahi et al. [24] and Panahi et al. [25]" please mention more details on these studies. Their sample. Their aim? "In addition, the results of the present study were not consistent with the results of the study by Vozikis et al. [15] " what was this study results and what is the controversy? | Both were done among students and we added to the relevant part.  Also, it is not appropriate to bring the goals in the discussion section. |
| In the discussion you could have compare the preventive behaviors in more detail with other studies. What type of behaviors were adapted the most? Which behaviors were neglected the most? It would be beneficial for future guidelines and educational purposes. | Thanks, but this case is not a part of the objectives of this study. |
| "The results also showed that gender was one of the factors affecting the adoption of preventive behaviors so the adoption of preventive behaviors was higher in female students than in male ones because following health principles, and medical recommendations, and more interest in learning and obtaining health information are more in women than in men." Reference please. | The reference was given. |
| " Mothers with higher education worked outside more and they go out more, thus their fear of COVID-19 was reduced. Therefore, they had probably fewer preventive behaviors and their children also followed their parents in adopting preventive behaviors." These statements are your opinion and your suggested reasons. But you have mentioned them like some facts. Please re-write this part or if they are proven in other studies please provide references. | It was revised. |
| Please provide a part on your suggestions or your studies implications. | Dear reviewer, we have mentioned these things at the end of the results section:  It is suggested to carry out more extensive studies to clarify the effect of health literacy on the adoption of preventive behaviors against COVID-19. Also, it seems necessary to design a special tool to measure health literacy related to COVID-19 in all age groups.  . |
