# Supplementary material for: The relationship between health literacy and the adoption of COVID-19 preventive behaviors: A cross-sectional study in Iran
Source: PLoS One. 2024 May 16;19(5):e0299007. doi: 10.1371/journal.pone.0299007 (PMC11098504; doi:10.1371/journal.pone.0299007)
Supplement: Attachment — Submitted filename: revision.docx [file pone.0299007.s007.docx]

**The highlighted comments were no addressed properly:**

Reviewer #6:
Line 55: you do not need to mention standard deviation. Just insert the mean score and insert SD in parentheses: 18.18 (4.02) Thanks a lot, it was revised.
Line 95: This seems more correct: University student population in Iran Thanks a lot, it was revised.
Line 96: "Due to the important role, they play as managers and future planners in the country" omit the comma Thanks a lot, it was revised.
Line 100: "Sajjadi et al.'s study showed that one-third of the students had insufficient or not very please for this and other studies mention the sample groups more specific. Thanks a lot, it was revised.
Line 102: " The critical situation of the prevalence of COVID-19 disease can cause negative and positive psycho-social effects in society including the university students [14]" this sentence seems unnecessary. what is the relevance of this to your study? Did you consider these effects in your work? Yes, we considered it.
We wanted to say that COVID-19 can even affect students.

Line 107-110: Was your sample group medical students or from other majors in health sciences? You may mention it here and it seems more reasonable when the participants are from health sciences. Esfrain Medical Sciences University has only 7 different fields of study including nursing, public health, intelligence, operating room technician, etc.
The method section was described thoroughly and in details. Some comments:
How did you chose the background variables?
We chose the background variables by studying various studies in the field of health literacy and COVID-19.
Line 113-116: "The statistical population was all the male and female students of Esfarayen Faculty of Medical Sciences, North Khorasan, Iran, among whom 214 were selected by simple random so that the names of all the students were listed and the participants were randomly selected." Please re-write this art in proper English writing.

The population included all male and female students at Esfarayen Medical Sciences University in Iran. The names of all students were listed and 214 participants were randomly selected by simple random sampling.
Line 131: parents' Thanks, it was revised.
Line 133: what do you mean by "type of COVID-19" We meant the type of infection, such as O’micron, Gamma, Beta, etc.

LINE 179: please insert numbers alphabetically in the beginning of a sentence. Ok
Table 1: what do you mean by "How to get content"- what content? It was revised.
Table 1: it would be much better if you asked participants the weekly amount of their exercise and in some ranges (e.g more than 2 hours, less than 2 hours …) this way it would be more objective.
Thanks a lot, your suggestion is very good, but the study has been completed and it can no longer be done.


Table 1: what is the importance of their Covid-19 type? What difference does it make? We wanted to see whether COVID-19 infection can be effective in preventing re-infection or initial infection.

Results: would you please a chart or graph (as supplementary items) that shows the questionnaires items and the frequency of each answers in specific?
Thanks so much, but the results should be mentioned based on the most important goals of the study.
Discussion:
" These results were consistent with the results of Panahi et al. [24] and Panahi et al. [25]" please mention more details on these studies. Their sample. Their aim?
"In addition, the results of the present study were not consistent with the results of the study by Vozikis et al. [15] " what was this study results and what is the controversy?
Both were done among students and we added to the relevant part.
Also, it is not appropriate to bring the goals in the discussion section.
In the discussion you could have compare the preventive behaviors in more detail with other studies. What type of behaviors were adapted the most? Which behaviors were neglected the most? It would be beneficial for future guidelines and educational purposes. Thanks, but this case is not a part of the objectives of this study.
"The results also showed that gender was one of the factors affecting the adoption of preventive behaviors so the adoption of preventive behaviors was higher in female students than in male
ones because following health principles, and medical recommendations, and more interest in learning and obtaining health information are more in women than in men." Reference please.
The reference was given.
" Mothers with higher education worked outside more and they go out more, thus their fear of COVID-19 was reduced. Therefore, they had probably fewer preventive behaviors and their children also followed their parents in adopting preventive behaviors." These statements are your opinion and your suggested reasons. But you have mentioned them like some facts. Please re-write this part or if they are proven in other studies please provide references. It was revised.
Please provide a part on your suggestions or your studies implications. Dear reviewer, we have mentioned these things at the end of the results section:
It is suggested to carry out more extensive studies to clarify the effect of health literacy on the adoption of preventive behaviors against COVID-19. Also, it seems necessary to design a special tool to measure health literacy related to COVID-19 in all age groups.
.
