# Supplementary material for: The relationship between health literacy and the adoption of COVID-19 preventive behaviors: A cross-sectional study in Iran
Source: PLoS One. 2024 May 16;19(5):e0299007. doi: 10.1371/journal.pone.0299007 (PMC11098504; doi:10.1371/journal.pone.0299007)
Supplement: Attachment — Submitted filename: Responses to the Reviewers(4th revised).docx [file pone.0299007.s008.docx]

| **Comments** | **Responses (The revised parts were highlighted in yellow inside the manuscript)** |
| --- | --- |
| Line 102: "The critical situation of the prevalence of COVID-19 disease can cause negative and positive psycho-social effects in society including the university students [14]" this sentence seems unnecessary. what is the relevance of this to your study? Did you consider these effects in your work? | Thanks a lot. It was revised and answered. (Fourth paragraph in the introduction part) |
| Results: would you please a chart or graph (as supplementary items) that shows the questionnaires items and the frequency of each answers in specific? | Dear reviewer; Within the methodology, we have determined that the Helia questionnaire had 33 items, the behavior questionnaire included 5 items, and 15 items belonged to the background demographic variables. As we have already answered, the results should be mentioned based on the most important objectives of the study. The case considered by the respected reviewer is not within the scope of our goals, because we believe that we have mentioned the most important findings based on the study goals. |
| Discussion:" **These results were consistent with the results of Panahi et al. [24] and Panahi et al. [25]"** please mention more details on these studies. Their sample. Their aim?  ……. "**In addition, the results of the present study were not consistent with the results of the study by Vozikis et al. [15]"** what was this study results and what is the controversy? Both were done among students and we added to the relevant part. Also, it is not appropriate to bring the goals in the discussion section.  …………… In the discussion you could have compare the preventive behaviors in more detail with other studies. What type of behaviors were adapted the most? Which behaviors were neglected the most? It would be beneficial for future guidelines and educational purposes. Thanks, but this case is not a part of the objectives of this study. | Thanks so much. It was revised and more details were brought into the manuscript.  Thanks so much. They were revised inside the manuscript. |
| "Mothers with higher education worked outside more and they go out more, thus their fear of COVID-19 was reduced. Therefore, they had probably fewer preventive behaviors and their children also followed their parents in adopting preventive behaviors."These statements are your opinion and your suggested reasons. But you have mentioned them like some facts. Please re-write this part or if they are proven in other studies please provide references. | Thanks so much. They were revised in the manuscript. |
| Please provide a part on your suggestions or your studies implications. | Dear reviewer:  we have mentioned these things at the end of the conclusion:  It is suggested to conduct more extensive studies to clarify the effect of health literacy on the adoption of preventive behaviors against COVID-19 In addition, it is recommended to investigate the relationship between different health literacy skills and the adoption of preventive behaviors against COVID-19 in future studies, so that more effective and shorter measures can be taken in this regard. Also, it seems necessary to design a special tool to measure health literacy related to COVID-19 among all age groups. |
